# Evaluating Hepatotoxicity: A Comparative Analysis of New Generation versus Historical Antiretroviral Agents

**DOI:** 10.3390/idr16030031

**Published:** 2024-04-24

**Authors:** Simona-Alina Abu-Awwad, Ahmed Abu-Awwad, Madalina-Ianca Suba, Voichita Elena Lazureanu, Andrei-Daniel Bolovan, Ovidiu Rosca, Mirela-Mădălina Turaiche, Adela-Teodora Benea, Bogdan Hogea

**Affiliations:** 1 Ist Clinic of Obstetrics and Gynecology, “Pius Brinzeu” County Clinical Emergency Hospital, 300723 Timisoara, Romania; alina.abuawwad@umft.ro; 2 Department of Obstetrics and Gynecology, Faculty of Medicine, “Victor Babes” University of Medicine and Pharmacy, 300041 Timisoara, Romania; 3 Department XV—Discipline of Orthopedics-Traumatology, “Victor Babes” University of Medicine and Pharmacy, 300041 Timisoara, Romania; ahm.abuawwad@umft.ro (A.A.-A.); hogea.bogdan@umft.ro (B.H.); 4 Research Center University Professor Doctor Teodor Sora, “Victor Babes” University of Medicine and Pharmacy, 300041 Timisoara, Romania; 5 Doctoral School, “Victor Babes” University of Medicine and Pharmacy, Eftimie Murgu Square, No. 2, 300041 Timisoara, Romania; andrei.bolovan@umft.ro (A.-D.B.); adela.benea@umft.ro (A.-T.B.); 6 Dr. Victor Babes, Infectious Diseases and Pneumophthisiology Hospital Timisoara, 300310 Timisoara, Romania; 7 Department XIII, Discipline of Infectious Diseases, “Victor Babes” University of Medicine and Pharmacy, Eftimie Murgu Square 2, 300041 Timisoara, Romania; lazureanu.voichita@umft.ro (V.E.L.); ovidiu.rosca@umft.ro (O.R.); 8 Department XV: Orthopedics-Traumatology, “Pius Brinzeu” Emergency Clinical County Hospital, Bld Liviu Rebreanu, No. 156, 300723 Timisoara, Romania; 9 Methodological and Infectious Diseases Research Center, Department of Infectious Diseases, “Victor Babes” University of Medicine and Pharmacy Timisoara, Eftimie Murgu Square 2, 300041 Timisoara, Romania; paliu.mirela@umft.ro

**Keywords:** HIV, antiretroviral therapy, liver metabolic health, hepatic metabolism, liver function, latest-generation ARV therapies, old-generation ARV therapies

## Abstract

(1) Background: Since the advent of zidovudine in 1987, antiretroviral therapy has undergone significant evolution, marked by the introduction of 34 antiretroviral drugs and 24 fixed-dose combinations. Despite these advances, hepatotoxicity remains a formidable challenge, influencing morbidity, mortality, and treatment adherence in HIV-infected patients. This study aims to compare the hepatotoxic effects of latest-generation antiretroviral medications with those of older-generation therapies, assessing their long-term impact on liver health in HIV patients. (2) Methods: This retrospective study analyzed data from 304 HIV patients treated with either latest-generation or older-generation antiretroviral drugs over four years. Patients were monitored for hepatotoxicity through liver function tests at diagnosis, six months, and one-year post-treatment initiation. (3) Results: Initial and six-month liver function tests showed no significant differences between the two groups. However, at one-year post-treatment, patients on latest-generation antiretrovirals exhibited significant improvements in ALT, AST, and ALP levels, suggesting a better safety profile regarding hepatotoxicity. Additionally, a significantly lower incidence of splenomegaly was observed in patients treated with newer medications. (4) Conclusions: The findings suggest that the latest-generation antiretroviral medications may offer a safer profile in terms of hepatotoxicity compared to older therapies, with potential benefits for long-term liver health. This study underscores the importance of continuous monitoring and further research to optimize ART strategies, ensuring improved patient outcomes and quality of life for individuals living with HIV.

## 1. Introduction

Since the initial introduction of the first antiretroviral drug, zidovudine, into clinical practice in 1987, the field of antiretroviral therapy has witnessed rapid advancements. The Food and Drug Administration (FDA) has subsequently sanctioned the approval of 34 distinct antiretroviral drugs, each distinguished by eight different mechanisms of antiviral action, along with the authorization of 24 fixed-dose combinations designed for the management of HIV infection [1]. Antiretroviral therapy has undergone a substantial transformation, progressing from therapeutic regimens burdened by a high number of pills, complex and inconvenient multiple daily dosing schedules, as well as treatment-limiting toxicities. It has subsequently entered a modern era characterized by the adoption of fixed-dose combinations and single-tablet regimens, facilitating the delivery of the complete treatment regimen through the administration of a solitary daily tablet [2].

Hepatotoxicity constitutes a notable and formidable complication observed in patients undergoing highly active antiretroviral therapy (HAART) [3]. Among the adverse effects attributed to the administration of antiretroviral drugs, the elevation of transaminases is noteworthy. Hepatic toxicity stands as a substantial contributor to morbidity, mortality, and the discontinuation of treatment in individuals afflicted with HIV infection [4]. Hepatotoxicity emerges as a potential risk element associated with suboptimal adherence to Highly Active Antiretroviral Therapy (HAART), thereby serving as a prominent catalyst for the development of drug-resistant viral strains. Additionally, subpar treatment adherence constitutes the principal risk factor contributing to virological and immunological failures, thereby complicating the decision-making process in the realm of medical management [5]. Presently, contemporary antiretroviral therapies exhibit a high degree of efficacy in the long-term maintenance of undetectable viral loads. However, it is imperative to acknowledge that drug toxicity, as well as the emergence and dissemination of drug resistance, continue to represent significant challenges that can hinder the overall success of therapy [6]. HIV infection alone can induce functional alterations in human liver sinusoidal endothelial cells (LSECs), which are responsible for the transportation of nutrients, lipids, and lipoproteins. These changes may lead to malfunctions characterized by decreased nitric oxide production and/or the under-expression of endothelial nitric oxide synthase (eNOS) [7].

Within the era of Highly Active Antiretroviral Therapy (HAART), the prevalence of hepatotoxicity exhibits a wide-ranging spectrum, spanning from 1% to 54.0% [8]. This phenomenon has been linked to the discontinuation of HAART, thereby significantly augmenting morbidity and mortality rates among individuals afflicted with HIV infection. A multitude of research studies have been conducted with the aim of elucidating the risk factors associated with hepatotoxicity in the context of HIV. These factors encompass variables such as age, gender, the specific HAART regimen utilized, viral load, the presence of hypertriglyceridemia and hyperglycemia, a documented history of tuberculosis therapy, concurrent infection with hepatitis B and hepatitis C, alcohol misuse, as well as elevated baseline levels of ALT or AST enzymes [9].

The purpose of this article is to assess and analyze the risk of hepatotoxicity among adult HIV-infected patients undergoing antiretroviral therapy with Bictegravir/Emtricitabine/Tenofovir Alafenamide (BIC/FTC/TAF) and Dolutegravir/Lamivudine (DTG/3TC) medications. By investigating and identifying potential hepatic adverse effects associated with these drugs, the aim is to provide a deeper understanding of the risks involved in antiretroviral treatment using these specific therapeutic agents. This research seeks to contribute to improving clinical approaches in managing HIV patients, offering essential insights for healthcare professionals to guide treatment decisions and minimize potential liver-related adverse effects in this vulnerable population.

## 2. Materials and Methods

### 2.1. Study Design and Setting

In this retrospective study, we conducted a comprehensive assessment of hepatotoxicity risk among adult HIV patients undergoing antiretroviral therapy (ART) with BIC/FTC/TAF and Dolutegravir/Lamivudine (DTG/3TC). The study was carried out at the “Dr. Victor Babeș, Clinical Hospital for Infectious Diseases and Pneumophthisiology” located in Timișoara, Romania. Over a four-year period, we retrospectively analyzed patient data to investigate the incidence and potential factors contributing to hepatotoxicity in this specific patient population. This medical center, equipped with advanced diagnostic facilities, provided an ideal setting for our research, and patients were routinely monitored for any adverse effects following the initiation of ART with BIC/FTC/TAF and DTG/3TC. The blood test results incorporated in this study are from three key time points: at the moment of diagnosis, six months after commencing treatment, and one year following the initiation of antiretroviral therapy.

### 2.2. Study Population

In this investigation, a total of 304 patients, all diagnosed with human immunodeficiency virus (HIV) infection, were included. We proceeded to form two distinct groups, namely Group 1 and Group 2, based on the type of antiretroviral therapy administered to the patients. Group 1 consisted of patients who exclusively received treatment with the antiretroviral medications BIC/FTC/TAF or DTG/3TC. On the other hand, Group 2 included patients who received treatment with older-generation antiretroviral drugs. This may encompass more traditional or older therapies that were utilized before the introduction of next-generation therapies.

This two-group approach allowed us to compare the effectiveness and tolerability of next-generation antiretroviral treatment, represented by BIC/FTC/TAF and DTG/3TC, with more traditional therapies. Thus, we assessed whether the new medications bring significant improvements regarding their hepatotoxicity effects.

To ensure the relevance and rigor of the study, strict inclusion criteria were established for the patients involved in the research. These criteria aim to select patients with suitable profiles for assessing the specific risk of hepatotoxicity associated with ART therapy using BIC/FTC/TAF and DTG/3TC.

Inclusion criteria:

- Confirmed diagnosis of human immunodeficiency virus 1 (HIV 1) infection.
- Adult age, 18 years or older.
- Patients who have received antiretroviral treatment for at least one year.
- Availability of relevant medical data for retrospective analysis.
- Patients who have not been previously diagnosed with preexisting liver disease.
- Patients who did not experience significant interruptions in their antiretroviral treatment during the study period.
- Patients who were regularly monitored for liver function and toxicity throughout their ART therapy.
- Patients who voluntarily consented to participate in the study and share the necessary medical data for retrospective analysis.

Exclusion criteria:

- Patients diagnosed with coexisting hepatitis B or C virus infection.
- Patients who did not consistently adhere to the dosing regimen of BIC/FTC/TAF and DTG/3TC medications. Non-adherence includes patients who did not regularly take their medications as prescribed, which could affect the effectiveness of the treatment and potentially lead to poorer health outcomes.
- Patients with a history of chronic alcoholism or substance abuse during ART treatment.
- Patients with unknown and unmonitored immunological status throughout the treatment.
- Patients with liver disease or renal failure. This is because liver conditions can influence the gastroenterological response of patients to treatment, just as renal failure can alter the response to antiretroviral therapy since many drugs are excreted or metabolized completely or partially by the kidneys.
- Pregnant or breastfeeding women, as different therapeutic regimens may be required in these cases.
- Patients who have previously participated in similar studies assessing hepatotoxicity with BIC/FTC/TAF and DTG/3TC.

### 2.3. Data Collection

To ensure the reliability and robustness of the study results, we implemented a systematic approach to data collection. The primary source of data was the hospital’s centralized electronic medical record (EMR) system, containing detailed patient profiles, including medical histories, medication regimens, laboratory test results, and documented adverse effects.

All collected data were anonymized to safeguard patient confidentiality and securely stored in an encrypted database with multi-level access controls, limiting access to authorized personnel only. Regular backups were performed to prevent data loss. Data extraction began in the month following the conclusion of the three-year study period and was completed within a three-month window to maintain data currency and relevance for analysis.

Given the retrospective nature of this study, we anticipated potential challenges such as missing records or inconsistent documentation. To address these issues, we established a protocol that involved consulting with healthcare providers involved in patient care or utilizing secondary sources like pharmacy records to validate and complete the dataset.

Careful planning and a systematic approach to data collection ensured that the gathered information was comprehensive and of high quality, laying the foundation for meaningful analysis in the subsequent stages of this study.

### 2.4. Statistical Analysis

For our statistical analyses in this study, we utilized GraphPad Prism 6 (GraphPad Software, Inc. Company, San Diego, CA, USA). We used two main statistical tests to determine the importance of the differences we observed in our data: the *t*-test and the Z-test. We selected the *t*-test when dealing with larger sample sizes and when our goal was to compare results between two distinct groups. On the other hand, we opted for the Z-test because our data were well-defined and standardized. We also employed the Z-test when we needed to compare two different proportions from the two groups, which were essentially two separate sets of data. This choice was made because the Z-test is more effective than the *t*-test for this type of data. The outcome of these tests is represented by the *p*-value, a numerical indicator. A *p*-value typically below 0.05 signifies statistically significant differences, demonstrating the importance of our findings.

To ensure the validity of our analysis, we used the Shapiro–Wilk test during data evaluation to verify the assumption of data normality, which is crucial in statistics. This test examined our data sample and confirmed that our data followed a normal distribution, making them suitable for the statistical methods we employed in our analysis.

In our analysis, the only dataset that exhibited non-normal distribution characteristics was the gamma-glutamyl transferase (GGT) values. To appropriately analyze these data, we calculated the median and interquartile range (IQR) instead of the mean and standard deviation, which are more suitable for normally distributed data. Given the skewed nature of the GGT values, we utilized the Mann–Whitney U test to calculate the *p*-value. This non-parametric test is ideal for comparing the central tendency of two independent groups when the data do not meet the assumptions of normality. By using this test, we were able to determine whether there were statistically significant differences between the two groups regarding their GGT levels.

### 2.5. Ethical Considerations

This study received approval from the Institutional Review Board at the hospital where it was conducted (approval No. 8947/28 September 2018). Since this was a retrospective study, the requirement for informed consent was waived. Nevertheless, we took strict measures to anonymize and manage all patient data in accordance with the General Data Protection Regulation (GDPR) guidelines, prioritizing patient confidentiality.

## 3. Results

In this medical study comparing two patient groups based on demographics, notable differences were observed in marital status and certain comorbid conditions (Table 1). Specifically, the latest generation group had a higher proportion of single, divorced, and widowed individuals, as well as increased incidences of diabetes and renal afflictions. In contrast, the older generation group had a greater number of patients without comorbidities. Gender, education, occupation, hypertension, and cardiovascular diseases, however, showed no significant differences between the groups. These findings highlight the diverse demographic and health profiles of patients undergoing different antiretroviral treatments.

Table 2 provides a comparative analysis of liver function tests between patients initiating treatment with the latest-generation antiretroviral medications (141 patients) and those with older-generation medications (163 patients). The tests include alanine aminotransferase (ALT), aspartate aminotransferase (AST), alkaline phosphatase (ALP), bilirubin levels, cholinesterase, and gamma-glutamyl transferase (GGT). 

Table 3 compares liver function test results between patients on the latest generation of antiretroviral medications (141 patients) and those on older-generation medications (163 patients) six months after starting treatment. The tests include ALT, AST, ALP, bilirubin levels, cholinesterase, and GGT. 

Table 4 compares liver function test results between two patient groups: those treated with the latest generation of antiretroviral medications (141 patients) and those with older-generation medications (163 patients) one year after starting the treatment.

The overall conclusion from the last three tables, which compare liver function test results between patients on the latest generation of antiretroviral medications and those on older-generation medications, is that there are no significant differences in most liver function parameters at the onset of treatment and at six months into the treatment. Both at the beginning and at the six-month mark, the liver function tests, including ALT, AST, ALP, bilirubin levels, cholinesterase, and GGT, show minimal or no statistically significant differences between the two groups. However, one year after starting treatment, there appears to be a notable improvement in liver function parameters such as ALT, AST, and ALP in patients on the latest generation antiretrovirals compared to those on older treatments. This suggests that while initial and short-term liver function is similar between the two groups, long-term liver function may be better in patients receiving the latest generation antiretroviral medications. Nevertheless, for other parameters like bilirubin, cholinesterase, and GGT, there remains no significant difference even after one year of treatment.

Table 5 presents ultrasound findings one year after initiating antiretroviral treatment, comparing patients treated with the latest generation of antiretroviral medications (141 patients) to those on older-generation medications (163 patients). The conditions assessed include splenomegaly, hepatomegaly, oral stomatitis, cholelithiasis (gallstones), cholecystitis, hepatic nodules, and hepatic steatosis. Notably, a significant difference is observed in the incidence of splenomegaly, with 4.96% in the latest generation group compared to 19.01% in the older generation group, indicating a significantly lower prevalence among those on newer medications (*p*-value: 0.0002). However, for other conditions like hepatomegaly, oral stomatitis, cholelithiasis, cholecystitis, hepatic nodules, and hepatic steatosis, the differences between the two groups are not statistically significant, as indicated by their respective *p*-values.

## 4. Discussion

This study embarked on an ambitious journey to explore the hepatotoxicity risk associated with the latest versus older-generation antiretroviral medications in 304 HIV patients, meticulously divided into two groups. 

Liver disease has been attributed to the hepatotoxic effects of various antiretroviral agents [10]. Subsequent to drug exposure, the toxic component initiates a cascade of stress responses or functional disturbances, with particular emphasis placed on the significance of mitochondrial injury as a recognized primary target [8]. The involvement of hepatocytes in metabolizing toxic drug substances renders them predisposed to drug-induced liver injury, leading to cellular demise as a consequential outcome [11]. Given its central role in the metabolism of a wide array of pharmaceutical agents, the liver often emerges as a prominent site susceptible to drug-induced injury, a phenomenon that extends to antiretroviral drugs [2].

Antiretroviral (ARV) medications have the capacity to induce harm to hepatic cells, either through direct action or via the influence of their active metabolites [12]. Conversely, within the context of HIV viral pathogenesis and liver injury, a myriad of mechanisms come into play, encompassing immune-mediated injury, oxidative stress, mitochondrial impairment, lipotoxicity, cytotoxicity, the accumulation of toxic metabolites, gut microbial translocation, and systemic inflammation [13].

The administration of antiretroviral therapy frequently encounters challenges due to the emergence of medication-associated side effects [14]. The division was predicated on the antiretroviral therapy received, with Group 1 receiving next-generation treatments like BIC/FTC/TAF or DTG/3TC and Group 2 on older-generation medications. 

Our demographic and clinical assessment unveiled nuanced differences in marital status and specific comorbid conditions, painting a complex picture of the health profiles influenced by antiretroviral therapies. At the time of diagnosis, six months later, and a year into the treatment, liver function tests—including alanine aminotransferase (ALT), aspartate aminotransferase (AST), alkaline phosphatase (ALP), bilirubin, cholinesterase, and gamma-glutamyl transferase (GGT) levels—were meticulously analyzed. The results, presented in Table 2 as mean values with standard deviations, show marginal differences in ALT, AST, ALP, and bilirubin levels between the two groups, with none of these differences reaching statistical significance, as indicated by their respective *p*-values (ALT: 0.551, AST: 0.079, ALP: 0.568, bilirubin: 0.662). Cholinesterase and GGT levels also do not show significant differences (*p*-values: 0.868 and 0.266, respectively). Overall, the table suggests that there are no significant differences in liver function test results at the onset of treatment between patients on the latest versus older-generation antiretroviral therapies.

Table 3 presents the results after three months of treatment. The mean values with standard deviations show that, at six months, the differences in ALT, AST, ALP, and bilirubin levels between the two groups are minimal and not statistically significant, as reflected in their *p*-values (ALT: 0.756, AST: 0.999, ALP: 0.459, bilirubin: 0.257). Similarly, there are no significant differences in cholinesterase and GGT levels (*p*-values: 0.469 and 0.370, respectively). Essentially, the table indicates that six months after the commencement of treatment, there are no significant disparities in these liver function parameters between patients on the latest and older-generation antiretroviral therapies.

Table 4 presents liver function test comparisons between patients on the latest and older-generation antiretroviral medications one year after starting the treatment. For alanine aminotransferase (ALT), the latest generation group has a lower mean level (29.17 ± 15.43) compared to the older generation (33.93 ± 22.37), with a statistically significant difference (*p*-value: 0.044). A similar pattern is observed with aspartate aminotransferase (AST), where the latest generation group shows a mean of 26.46 ± 10.83, significantly lower than the 42.37 ± 66.04 of the older generation (*p*-value: 0.005). Alkaline phosphatase (ALP) levels also differ, with the latest generation group showing a lower mean (100.3 ± 45.56) compared to the older generation (114.4 ± 69.80, *p*-value: 0.035). However, bilirubin levels, cholinesterase, and gamma-glutamyl transferase (GGT) do not show statistically significant differences between the two groups, as indicated by their respective *p*-values (0.285, 0.483, and 0.762). This suggests that patients on the latest generation antiretrovirals may have better liver function profiles regarding ALT, AST, and ALP but similar bilirubin, cholinesterase, and GGT levels compared to those on older antiretrovirals.

Patients whose transaminase levels (ALT and AST) are normal at the start of treatment are considered to have developed hepatotoxicity if their ALT and/or AST levels rise above the normal upper limit during their antiretroviral therapy. It is crucial to recognize that various medications can cause an increase in gamma-glutamyl transpeptidase (GGT) levels. In our study, there were no statistically significant differences between the GGT levels of patients from both groups at the measured time intervals. However, this increase should not always be interpreted as a sign of liver damage; it might merely signify that the enzyme is more active [15]. Concerns regarding potential liver issues should arise only if there is a simultaneous increase in alkaline phosphatase levels, which could indicate a possible obstruction in bile flow [16]. Also, elevated bilirubin levels, by themselves, are not a dependable marker of liver toxicity, as they can be affected by a range of factors, including specific health conditions, fasting, and particular medications like indinavir and atazanavir [17].

In addition to hepatotoxicity that may result from highly active antiretroviral therapy, other conditions or medications linked to HIV infection could also cause an elevation in liver enzyme levels [4]. This is why we included in our exclusion criteria patients who have other pathologies or are undergoing other treatments that could affect liver function.

In recent years, the mortality rate associated with HIV and the occurrence of opportunistic infections have significantly declined, thanks to the advent of highly active antiretroviral therapy [18]. This therapy strategy involves the use of a combination of two or more antiretroviral drugs, commonly referred to as antiretroviral therapy [19].

Initially, and at the six-month mark, the differences between the groups in these liver function parameters were minimal and statistically insignificant. This finding underscores a critical insight: the newer antiretroviral therapies, at their onset and halfway through the first year, do not adversely affect liver function more or less than their older counterparts. However, one year after starting the treatment, significant differences are observed in the increase in transaminases and ALP. Through these results, we reinforce the findings of other studies [20,21,22] that demonstrate that the new generation therapy affects liver function less than the older generation therapy.

A notable decline in liver function parameters (ALT, AST, and ALP) was observed in Group 2, indicating a potential long-term benefit of newer antiretroviral medications in preserving liver health. It was demonstrated that transitioning to the latest generation of antiretroviral medications was linked with significant viral suppression, better lipid profile outcomes, and the prevention of drug–drug interactions in a substantial segment of this real-world cohort of older people living with HIV [23].

This improvement did not extend to bilirubin, cholinesterase, and GGT levels, which remained comparably unaffected across both groups, suggesting that the advantages of newer therapies might be specific to certain liver function aspects.

However, some studies have demonstrated that in antiretroviral therapy, all liver enzymes are elevated [24], while others show that only transaminases are elevated in this therapy [25].

Patients on the latest generation antiretroviral medications exhibit a significantly lower incidence of splenomegaly compared to those on older-generation medications one year after treatment initiation, suggesting a possible advantage of newer antiretrovirals in reducing certain complications.

The similarity in the prevalence of conditions such as hepatomegaly, oral stomatitis, cholelithiasis, cholecystitis, hepatic nodules, and hepatic steatosis between the two groups suggests that the latest generation antiretrovirals do not increase the risk of these specific ultrasound-detected abnormalities compared to older medications.

Ultrasound findings added another layer of depth to our understanding. A significantly lower incidence of splenomegaly in Group 1 versus Group 2, a year into treatment, hinted at the possible benefits of newer medications beyond liver enzyme levels. Yet, for other conditions assessed by ultrasound, including hepatomegaly, oral stomatitis, and cholelithiasis, the incidence rates did not significantly diverge, indicating that the risk for these conditions remains uniformly distributed regardless of the antiretroviral generation. In patients with HIV infection, a study demonstrated hepatobiliary disease in 22.8% of cases [26]. The clinical presentation was asymptomatic in the majority of cases, similar to patients with hepatobiliary involvement in our study.

The study demonstrates that the newer antiretroviral medications might offer a safer profile concerning hepatotoxicity risk over the long term, as evidenced by improved ALT, AST, and ALP levels without compromising efficacy. The presence of comorbid conditions across both treatment groups underscores the need for comprehensive care strategies tailored to the complex health profiles of HIV patients, integrating considerations for mental health, chronic diseases, and lifestyle counseling.

The findings pave the way for future research to explore the mechanisms behind the improved liver function outcomes associated with newer antiretrovirals and to assess their long-term impact on patient quality of life and treatment adherence.

The study reinforces the importance of a holistic approach in managing HIV, where treatment decisions are informed by a thorough understanding of the potential side effects, patient demographics, and the evolving landscape of antiretroviral therapy.

### Strengths and Limitations

This study’s strengths lie in its comparative design, which directly assesses the hepatotoxic effects of the latest versus older-generation antiretroviral medications in a sizable cohort of 304 HIV patients, providing valuable insights into the long-term safety profiles of these treatments. The inclusion of diverse liver function tests and ultrasound findings adds a comprehensive evaluation of potential hepatotoxicity, enhancing the study’s clinical relevance. Additionally, the longitudinal approach, with assessments at baseline, six months, and one year, allows for a nuanced understanding of the temporal changes in liver health attributable to these therapies.

However, the study faces limitations, including its observational nature, which, while effective for detecting associations, cannot definitively establish causality between the type of antiretroviral medication and observed liver function changes. The lack of randomization may introduce selection bias, potentially affecting the comparability of the two groups. Furthermore, the study’s reliance on liver function tests and ultrasound findings, without corroborating these with liver biopsies or more sensitive imaging techniques, might limit the accuracy in diagnosing and grading liver conditions. Lastly, the demographic and clinical diversity of the patient population, while reflective of real-world scenarios, complicates the generalization of findings across different HIV patient subgroups.

## 5. Conclusions

This study provides a comprehensive comparison of the hepatotoxic effects associated with the latest versus older-generation antiretroviral medications in HIV patients. Key findings highlight that, although no significant differences in liver function were observed initially or at six months, patients on newer antiretrovirals demonstrated significant improvements in liver function tests, such as ALT, AST, and ALP, one year after treatment initiation. This suggests a potentially enhanced safety profile for newer medications regarding liver health. Additionally, a reduced incidence of splenomegaly in the group receiving the latest generation treatments points to their benefit in minimizing certain liver-related complications. Despite its observational nature and potential biases, the study significantly contributes to understanding the long-term impacts of antiretroviral therapy on liver health, advocating for ongoing monitoring and further research to optimize HIV treatment strategies.

## Figures and Tables

**Table 1:** Demographic and clinical profile comparison between patients on latest and older generation antiretroviral therapies.

Demographic Criteria	Group 1 (N = 141)	Group 2 (N = 163)	*p* Value
Gender:			
- Male	86 (60.99%)	91 (55.82%)	0.362
- Female	55 (39%)	72 (44.17%)	0.362
Marital Status:			
- Married	30 (21.27%)	28 (17.17%)	0.365
- Single	84 (59.57%)	69 (42.33%)	0.002
- Divorced	21 (14.89%)	47 (28.83%)	0.003
- Widowed	6 (4.25%)	19 (11.65%)	0.019
Education:			
-No formal education	9 (6.38%)	13 (7.97%)	0.594
- High School	61 (43.26%)	69 (42.33%)	0.870
-Bachelor′s/College Degree	59 (41.84%)	71 (43.55%)	0.764
-Post-graduate Studies	12 (8.51%)	10 (6.13%)	0.425
Occupation:			
- Unemployed	17 (12.05%)	25 (15.33%)	0.409
- Skilled Worker	46 (32.62%)	62 (38.03%)	0.326
- Professional	51 (36.17%)	52 (31.90%)	0.433
- Retiree	27 (19.14%)	24 (14.72%)	0.304
Comorbidities:			
- Hypertension	14 (9.92%)	21 (12.88%)	0.420
- Diabetes	21 (14.89%)	8 (4.90%)	0.003
-Cardiovascular Diseases	9 (6.38%)	13 (7.97%)	0.594
- Renal Afflictions	11 (7.80%)	3 (1.84%)	0.007
-Without Comorbidities	86 (60.99%)	118 (72.39%)	0.035

**Table 2:** Liver function test comparisons between patients on the latest and older generation antiretroviral medications at the time of starting the treatment.

	Group 1 (N = 141)	Group 2 (N = 163)	*p* Value
ALT (U/L)			
Mean ± SD	29.99 ± 18.17	31.34 ± 19.34	0.551
AST (U/L)			
Mean ± SD	26.37 ± 10.05	29.13 ± 16.07	0.079
ALP (U/L)			
Mean ± SD (IU/L)	99.21 ± 45.79	102.8 ± 63.42	0.568
Bilirubin levels			
Mean ± SD (mg/dL)	0.6714 ± 0.3248	0.6867 ± 0.2793	0.662
Cholinesterase (U/L)			
Mean ± SD	7874 ± 2169	7829 ± 2572	0.868
	Median: 51	Median: 57	
GGT (U/L)	IQR: 39	IQR: 37	0.266

**Table 3:** Liver function test comparisons between patients on the latest and older generation antiretroviral medications six months after starting the treatment.

	Group 1 (N = 141)	Group 2 (N = 163)	*p*-Value
ALT (U/L)			
Mean ± SD	30.91 ± 18.76	31.63 ± 19.52	0.756
AST (U/L)			
Mean ± SD	28.79 ± 11.65	28.79 ± 15.18	0.999
ALP (U/L)			
Mean ± SD	97.21 ± 45.56	103.9 ± 63.31	0.459
Bilirubin levels (mg/dL)			
Mean ± SD	0.6649 ± 0.3126	0.7036 ± 0.2796	0.257
Cholinesterase (U/L)			
Mean ± SD	7996 ± 2086	7804 ± 2484	0.469
GGT (U/L)	Median: 51	Median: 51	
	IQR: 39	IQR: 42	0.370

**Table 4:** Liver function test comparisons between patients on the latest and older generation antiretroviral medications one year after starting the treatment.

	Group 1 (N = 141)	Group 2 (N = 163)	*p*-Value
ALT (U/L)			
Mean ± SD	29.17 ± 15.43	33.93 ± 22.37	0.044
AST (U/L)			
Mean ± SD	26.46 ± 10.83	42.37 ± 66.04	0.005
ALP (U/L)			
Mean ± SD	100.3 ± 45.56	114.4 ± 69.80	0.035
Bilirubin levels (mg/dL)			
Mean ± SD	0.6480 ± 0.3396	0.6852 ± 0.2616	0.285
Cholinesterase (U/L)			
Mean ± SD	7873 ± 2162	7682 ± 2542	0.483
GGT (U/L)	Median: 56	Median: 51	
	IQR: 38	IQR: 43	0.762

**Table 5:** Ultrasound findings one year after initiating antiretroviral treatment: a comparison between the latest and older generation medications.

Results	Group 1 (N = 141)	Group 2 (N = 163)	*p* Value
Splenomegaly	7 (4.96%)	31 (19.01%)	0.0002
Hepatomegaly	9 (6.38%)	11 (6.74%)	0.899
Oral stomatitis	13 (9.21%)	24 (14.72%)	0.143
Cholelithiasis (gallstones)	13 (9.21%)	9 (5.52%)	0.216
Cholecystitis	7 (4.96%)	7 (4.29%)	0.781
Hepatic nodules	3 (2.12%)	9 (5.52%)	0.129
Hepatic steatosis	17 (12.05%)	21 (12.88%)	0.827

## Data Availability

The datasets used and/or analyzed during the current study are available from the first author.
